# 1983. Clinical outcomes of people living with HIV and cirrhosis: a clinical cohort study

**DOI:** 10.1093/ofid/ofad500.110

**Published:** 2023-11-27

**Authors:** Elizabeth C Arant, Cynthia L Gay, Thibaut Davy-Mendez, Andrew M Moon

**Affiliations:** University of North Carolina at Chapel Hill, Chapel Hill, NC; UNC-Chapel Hill, Chapel Hill, North Carolina; UNC Chapel Hill, Chapel Hill, North Carolina; The University of North Carolina at Chapel Hill, Chapel Hill, North Carolina

## Abstract

**Background:**

People with HIV (PWH) are aging, with an increasing prevalence of cirrhosis from causes such as viral hepatitis, non-alcoholic fatty liver disease (NAFLD) and alcohol-associated liver disease (ALD). It is hypothesized that HIV could accelerate the progress of liver disease, but the characteristics and outcomes of PWH with cirrhosis are not well-known. We characterized patient characteristics and longitudinal outcomes of PWH with cirrhosis in a clinical setting.

**Methods:**

This was a retrospective cohort study including PWH ≥18 years old with cirrhosis in HIV care in the UNC Center for AIDS Research HIV Clinical Cohort between 2014 and 2022. We estimated incidence rates (IR) of hospitalization, hepatocellular carcinoma (HCC), and death. In a subgroup of PWH with incident cirrhosis, we estimated 3-year cumulative incidence (CI) of first decompensation event and death from Kaplan Meier curves.

**Results:**

The 114 PWH with cirrhosis were mostly male (78%), Black (53%), aged 50-59 (44%), and 38% had Medicare. A third (32%) had injection drug use (IDU) as the mode of HIV transmission, and almost all were virally suppressed (91%). Hepatitis C virus infection was the most common cirrhosis etiology (62%), followed by ALD (24%). Unadjusted IR per 100 person-years was 27.0% (95% CI 20.2-36.1) for hospitalization, 1.8% (95% CI 1.1-3.1) for HCC, and 3.8% (95% CI 2.6-5.6) for death. Among 69 PWH with incident cirrhosis, the 3-year CI was 38.0 (95% CI 32.0-44.0) for decompensation and 10.9 (95% CI 7.0-14.9) for death (Fig. 1). Three participants (2.6%) were referred for liver transplantation during the study period, however none received a transplant.

Figure 1

**Figure:**
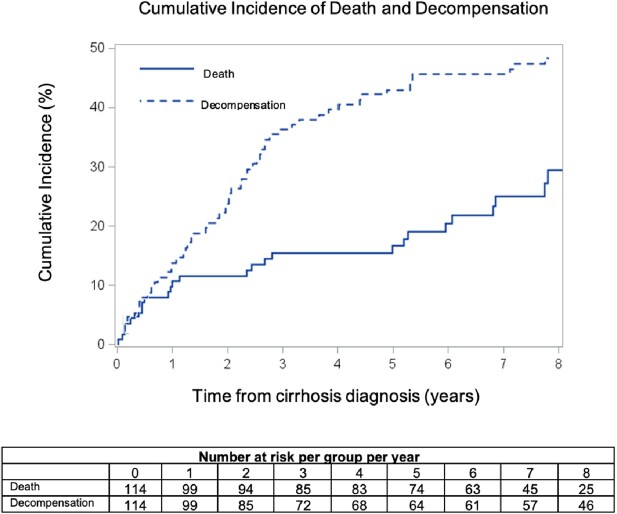

**Conclusion:**

PWH with cirrhosis in this cohort had high rates of adverse health outcomes, with hospitalization and death rates that are higher than those reported among PWH overall. Despite being engaged in HIV care, over a third of PWH experienced decompensation within 3 years, indicating a need for earlier consideration for liver transplantation. Analyses are ongoing to compare clinical outcomes by cirrhosis etiology and severity at diagnosis.

**Disclosures:**

**Andrew M. Moon, MD, MPH**, TARGET RWE: Advisor/Consultant

